# Combined effect of the pro-apoptotic rhTRAIL protein and HSV-1 virus in head and neck cancer cell lines

**DOI:** 10.1038/s41598-023-44888-9

**Published:** 2023-10-21

**Authors:** Lucas Bravo Perina, Izabela Natalia Faria Gomes, Ana Rúbia Alcantara Pelloso, Viviane Aline Oliveira Silva, Lidia Maria Rebolho Batista Arantes, Matias Eliseo Melendez

**Affiliations:** 1 Molecular Oncology Research Center, Barretos Cancer Hospital, Barretos, SP 14784-400 Brazil; 2 Molecular Carcinogenesis Program, National Cancer Institute (INCA), Rio de Janeiro, RJ 20230-240 Brazil; 3 Department of Pathology and Legal Medicine, Medical School of the Federal University of Bahia, Salvador, BA 40026-010 Brazil; 4 Laboratory of Pathology and Molecular Biology, Gonçalo Moniz Institute, Oswaldo Cruz Foundation, Salvador, BA 40296-710 Brazil

**Keywords:** Oncology, Cancer therapy, Head and neck cancer

## Abstract

Knowledge on the molecular and clinical characteristics of head and neck squamous cell carcinoma (HNSCC) is vast. However, an effective therapy that increases the life expectancy of these patients, with a 5-year overall survival of 50%, is still unknown. Here we evaluated the combined effect of the pro-apoptotic protein rhTRAIL with the replication-competent wild-type HSV-1 virus in head and neck cancer cell lines. We observed a difference in the modulation profile of proteins related to apoptotic pathways in the studied cell lines. The HCB289 exhibited caspase-9 activation in the presence of the HSV-1 virus, while the UD-SCC-2 exhibited caspase-8 activation in the presence of rhTRAIL. Both cell lines exhibited PARP activation by combining rhTRAIL and HSV-1 virus treatment. Flow cytometry analysis exhibited greater induction of late apoptosis for the HCB289 and UD-SCC-2 after the combination treatment of the HSV-1 and rhTRAIL. However, the UD-SCC-2 also presented induction of late apoptosis by the presence of rhTRAIL in monotherapy. These data suggest an enhancement of the effect of the combination treatment of the rhTRAIL and the HSV-1 on reducing viability and induction of cell death.

Head and neck cancer is a group of tumors affecting the upper aerodigestive epithelium, mainly involving the lips, larynx, pharynx, paranasal sinuses, nasal cavity, and oral cavity^1^_._ In 90% of cases, head and neck squamous cell carcinoma (HNSCC) appears to be the most common morphology of the other histological types^1^. Worldwide, HNSCC has an incidence of 650,000 and a mortality rate of 330,000 new cases per year, with the highest incidence and mortality of cancer of the lips and oral cavity^2^. Men have a higher frequency of cases than women, with a ratio ranging from 2:1 to 4:1^3^.

Because 20–30% of patients do not respond to standard treatments due to local recurrence, alternative medicines for the treatment of HNSCC have been developed, some of which have previously passed animal testing and are now in the clinical trial phase^4, 5^. Among them, oncolytic gene therapy (based on replicating competent viruses) has shown promising results in HNSCC. Most HNSCC tumors do not develop distant metastasis and the local/regional nature of HNSCC tumors facilitates the application of intratumoral injections^6–8^. Such characteristic favor local treatment and avoid systemic dissemination of the therapeutic vector. In addition, herpes simplex virus type 1 (HSV-1)-derived oncolytic viruses have already been evaluated in clinical trials and Talimogene laherparepvec (T-VEC), a genetically modified virus from HSV-1, has already been approved by the Food and Drug Administration (FDA) for the treatment of melanoma^9, 10^. The T-VEC was analyzed for HNSCC in one study of phase Ib in combination of pembrolizumab, however the combination has an efficiency similar to pembrolizumab alone compare to other historical HNSCC studies and the phase III did not proceed^11^.

Improvement of oncolytic virotherapy could be achieved by cell death induction, combining its use with pro-apoptotic recombinant proteins. TNF-related apoptosis-inducing ligand (Apo2L/TRAIL protein) has been widely explored in due to its specificity in inducing apoptosis in tumor cells without affecting healthy cells^12–16^. Apo2L/TRAIL triggers apoptosis by binding to functional death receptors (DR4 and DR5)^17, 18^, but, regardless of the fact that these receptors are highly expressed in HNSCC lines, many tumor cell lines end up showing resistance to treatment with TRAIL monotherapy^18, 19^.

Several TRAIL receptor agonists are being evaluated in clinical trials, such as the rhTRAIL ligand and monoclonal antibodies (mapatumumab, lexatumumab, tigatuzumab, apomab, conatumumab, and drozitumumab)^12, 20–23^. Studies on TRAIL ligand agonists have shown that, although they can induce cell death in sensitive tumors, they need to be combined with other therapies to increase their efficacy and utility in primary tumors^12–14, 16^.

Thus, combining both therapeutic approaches, oncolytic virotherapy and TRAIL-mediated apoptosis, may be promising in for HNSCC treatment. In this work, we evaluated the combined treatment of rhTRAIL protein and replication-competent wild-type HSV-1 virus in HNSCC cell lines.

## Results

### Combination of rhTRAIL and HSV-1 decrease cell viability in HNSCC cell lines

**Figure 1:**
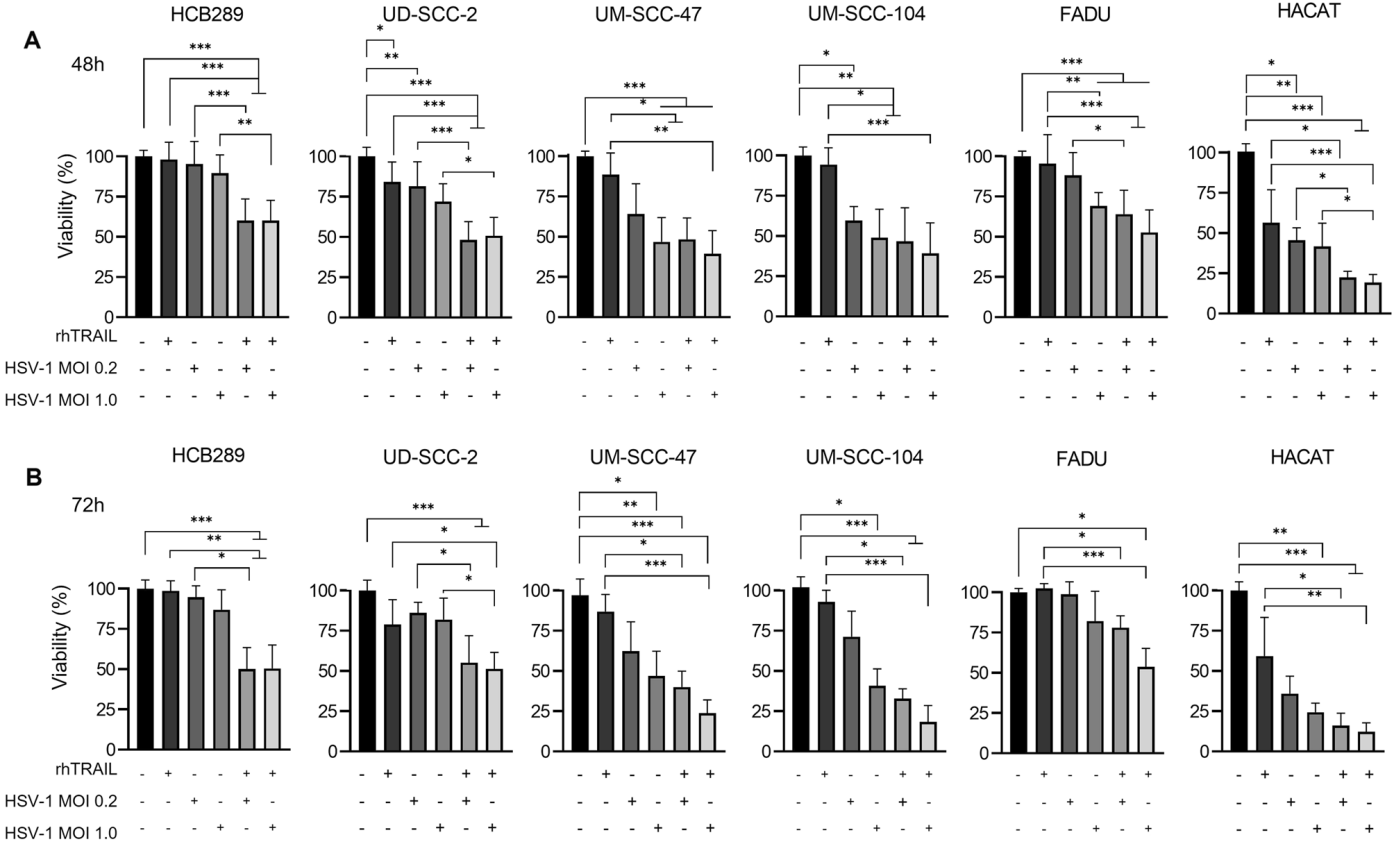
rhTRAIL exposure effect alone and in combination with WT HSV-1 virus on HNSCC cell lines and the normal cell line, HaCaT. Treatment was performed with rhTRAIL at 100 ng/mL and in combination with WT HSV-1 virus at MOI 0f. 0.2 and 1.0. Cell viability analysis after 48 (**A**) and 72 h (**B**) of exposure. Results are expressed by mean percentage ± standard deviation of cell viability relative to PBS 1X (considering 100% viability). Data represent the average of three independent assays performed in triplicate. The asterisks indicate statistical significance (*P < 0.05; **P < 0.01; ***P < 0.001) in the comparison with the experimental groups by non-normal distribution Kruskal–Wallis test.

Five HNSCC (UD-SCC-2; UM-SCC-47; UM-SCC-104, FaDu, and HCB289) and an immortalized human keratinocytes (HaCaT) cell lines were treated with rhTRAIL ligand and/or wt-HSV-1 virus for 48 and 72 h (Fig. 1). rhTRAIL resistance threshold was set in 100 ng/mL^24^.

After 48 h of treatment, cell lines showed a decrease in cell viability when treated with WT HSV-1 at MOI 1 alone or in combination with rhTRAIL. HaCat also showed a decrease in cell viability of 22.47%, when treated with HSV-1 at MOI 0.2 (Fig. 1A). For 72 h, we observed the HCB289, UD-SCC2 and FaDu showed a significant effect upon exposure to the combination of rhTRAIL and WT HSV-1. Although we used the HaCat as a normal control and the rhTRAIL was previously described as promoting apoptosis mainly tumor cells, it was affected by the rhTRAIL treatment, which was not expected. The UM-SCC-47, UM-SCC-104 and HaCaT, also showed a decrease in viability when exposed to the virus alone (Fig. 1B).

### HCB289 and UD-SCC-2 has different modulation pattern of proteins associated with the apoptotic pathway

To characterize the possible mechanisms responsible for cell viability decreases in HNSCC cell lines, we investigated the protein expression modulation associated with intrinsic and extrinsic apoptotic pathways after 24 and 48 h of exposure to rhTRAIL and its combination with the WT HSV-1. We selected the HCB289 and UD-SCC-2 for a cell death function characterization. This selection was made from the observation that for both, 48 and 72 h, these HNSCC cell lines showed a significant reduction in cell viability to exposure to the combination rhTRAIL and virus compared to rhTRAIL or virus as monotherapy.

**Figure 2:**
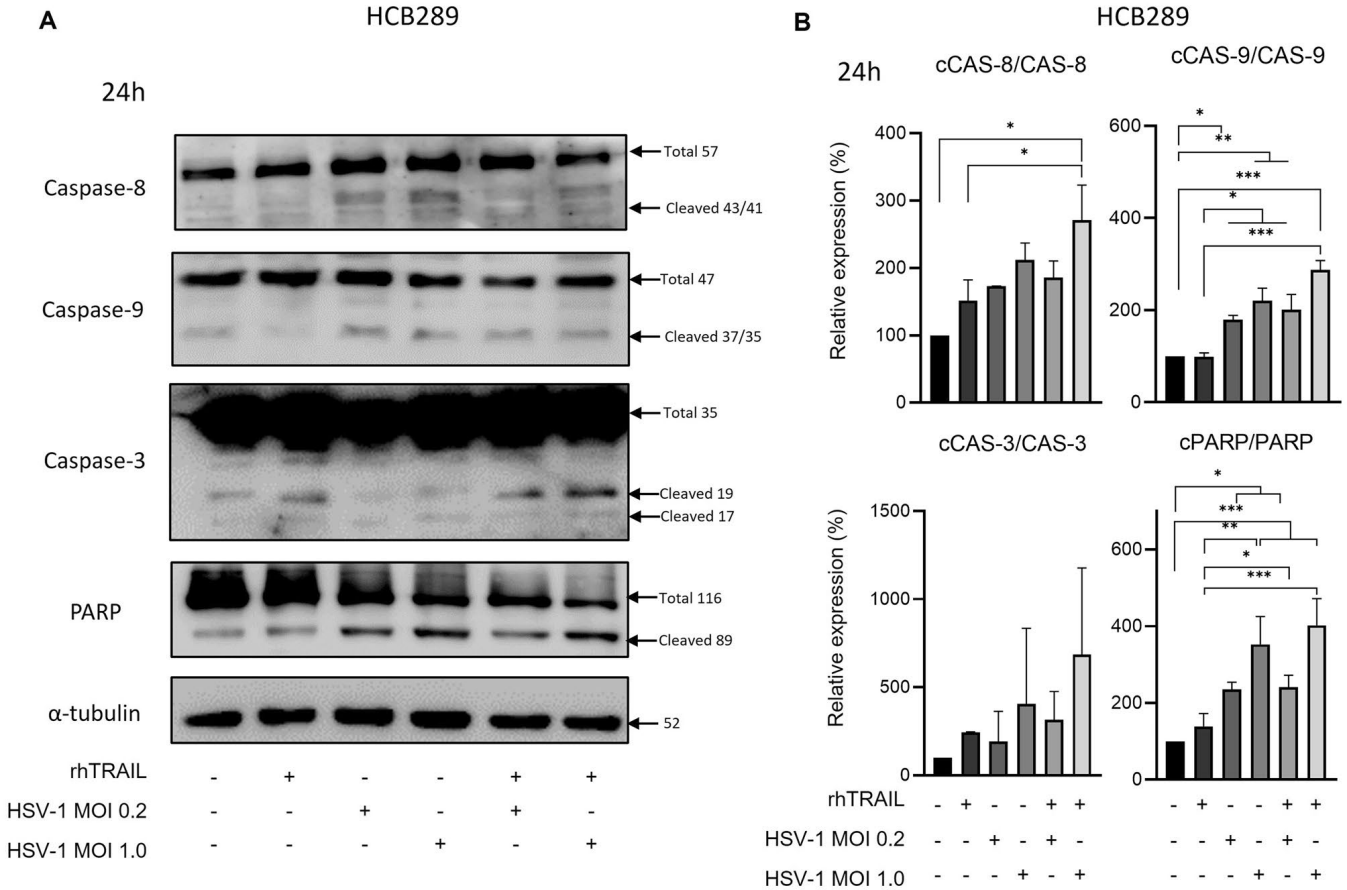
Expression analysis of apoptosis-associated proteins in the HCB289 cell line after 24 h of exposure with rhTRAIL ligand, WT HSV-1 and in combination. (**A**) and (**B**) detection and quantification of proteins after treatment. Results are expressed as mean percentage ± standard deviation of relative expression of cleaved protein relative to control (considering 100% expression) and normalized by the portion of total protein (non-cleaved). Data represent the average of three independent assays. The asterisks indicate statistical significance (*P < 0.05; **P < 0.01; ***P < 0.001) in the comparison with the experimental groups by one-way ANOVA. Raw blots are presented in Supplementary Data 1.

Results for HCB289 showed modulation on caspase-9 and PARP cleavages after 24 h of exposition with the combination of rhTRAIL and virus, especially under conditions of the virus at MOI 1.0 of 287.63% and 402% relative expression respectively compared to negative control. However, the effect on caspase-8 cleavage was evident only with the rhTRAIL and HSV-1 MOI 1 combination (2.7 times higher than control group). On the other hand, we did not observe a significant change in the caspase-3 cleavage under different treatment conditions (Fig. 2A,B).

**Figure 3:**
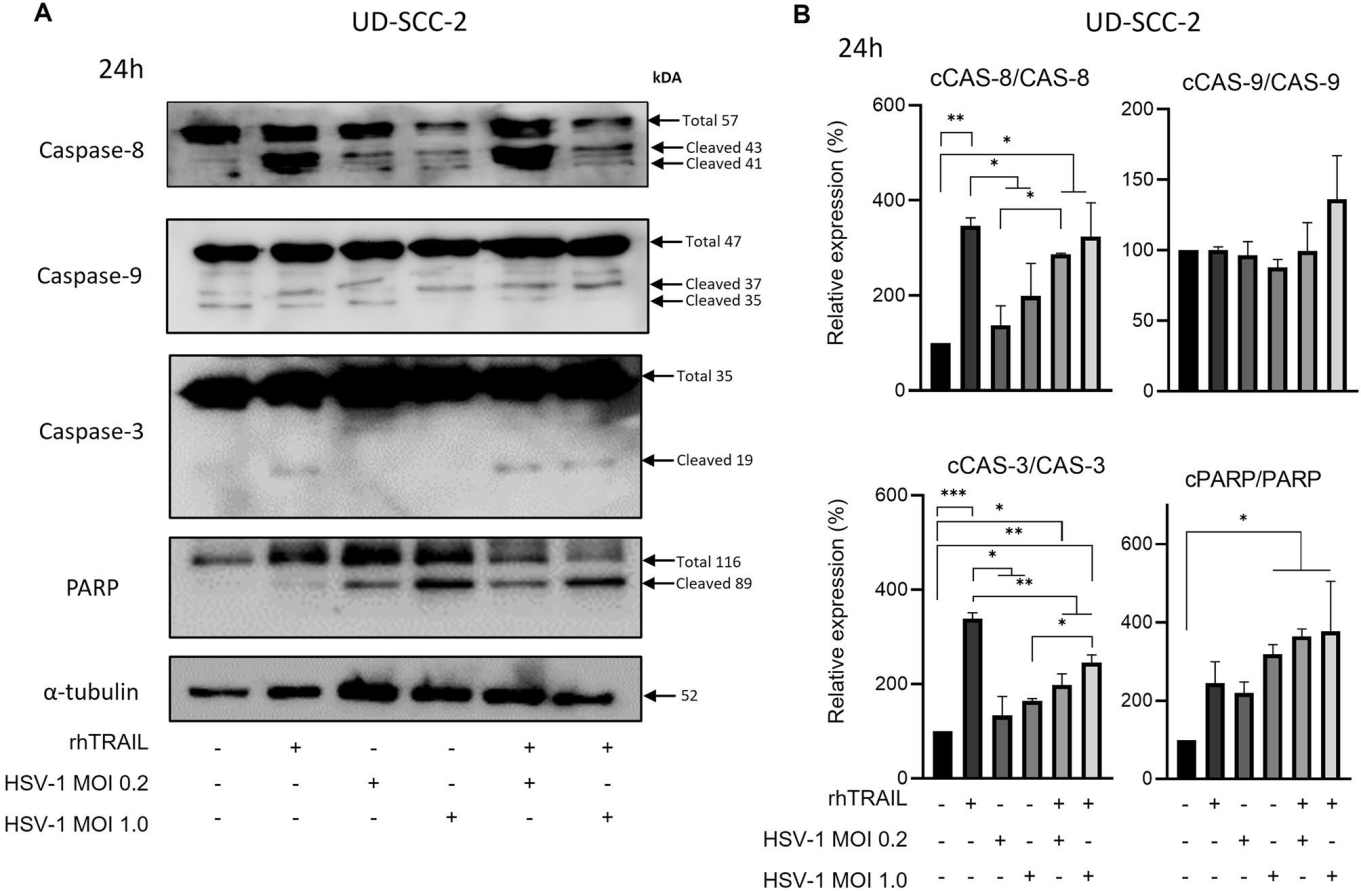
Expression analysis of apoptosis-associated proteins in the UD-SCC-2 cell line after 24 h of exposure with rhTRAIL ligand, WT HSV-1 and in combination. (**A**) and (**B**) detection and quantification of proteins after treatment. Results are expressed as mean percentage ± standard deviation of relative expression of cleaved protein relative to control (considering 100% expression) and normalized by the portion of total protein (non-cleaved). Data represent the average of three independent assays. The asterisks indicate statistical significance (*P < 0.05; **P < 0.01; ***P < 0.001) in the comparison with the experimental groups by one-way ANOVA. Raw blots are presented in Supplementary Data 2.

For UD-SCC-2, the 24 h exposition treatment promoted alterations on incrementation of caspase-8 cleavage when exposed to rhTRAIL alone (346.92%) or in combination with HSV-1 at MOI 0.2 (286.84%) or 1.0 (324.09%). Besides, PARP cleavage significantly increases at HSV-1 presence at MOI 1.0 (3.19 times higher) and in combination with rhTRAIL on both concentrations (3.65 and 3.77 times higher). Increased Caspase-3 cleavage was reported at rhTRAIL monotherapy (3.38 times higher) and in the combination of HSV-1 at MOI 1.0 (2.45 times higher) compared to negative control. No differences were detected for caspase-9 cleavage under different treatment conditions (Fig. 3A,B).

**Figure 4:**
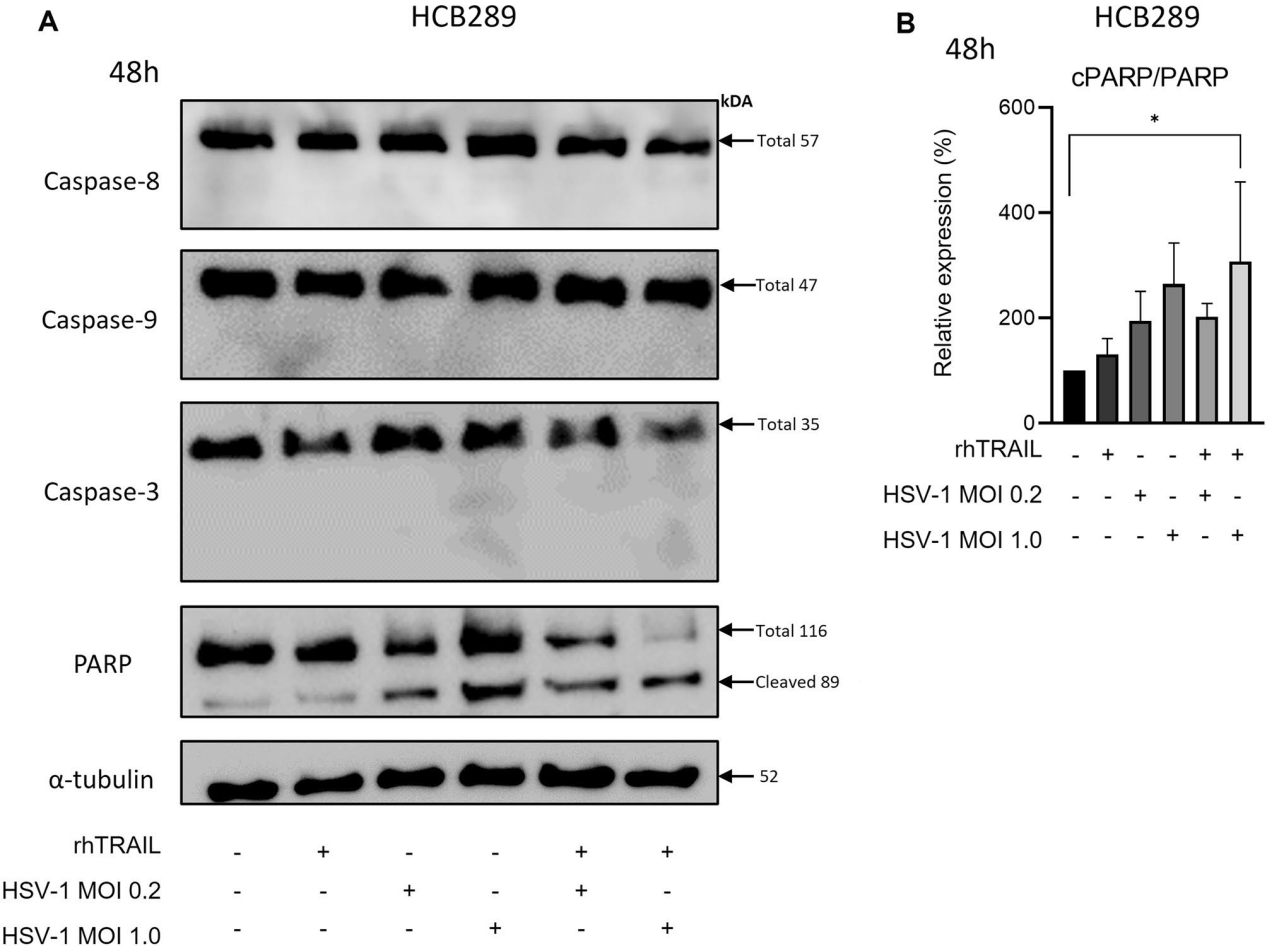
Expression analysis of apoptosis-associated proteins in the HCB289 cell line after 48 h of exposure with rhTRAIL ligand, WT HSV-1 and in combination. (**A**) and (**B**) detection and quantification of proteins after treatment. Results are expressed as mean percentage ± standard deviation of relative expression of cleaved protein relative to control (considering 100% expression) and normalized by the portion of total protein (non-cleaved). Data represent the average of two independent assays. The asterisks indicate statistical significance (*P < 0.05) in the comparison with the experimental groups by one-way ANOVA. Raw blots are presented in Supplementary Data 3.

**Figure 5:**
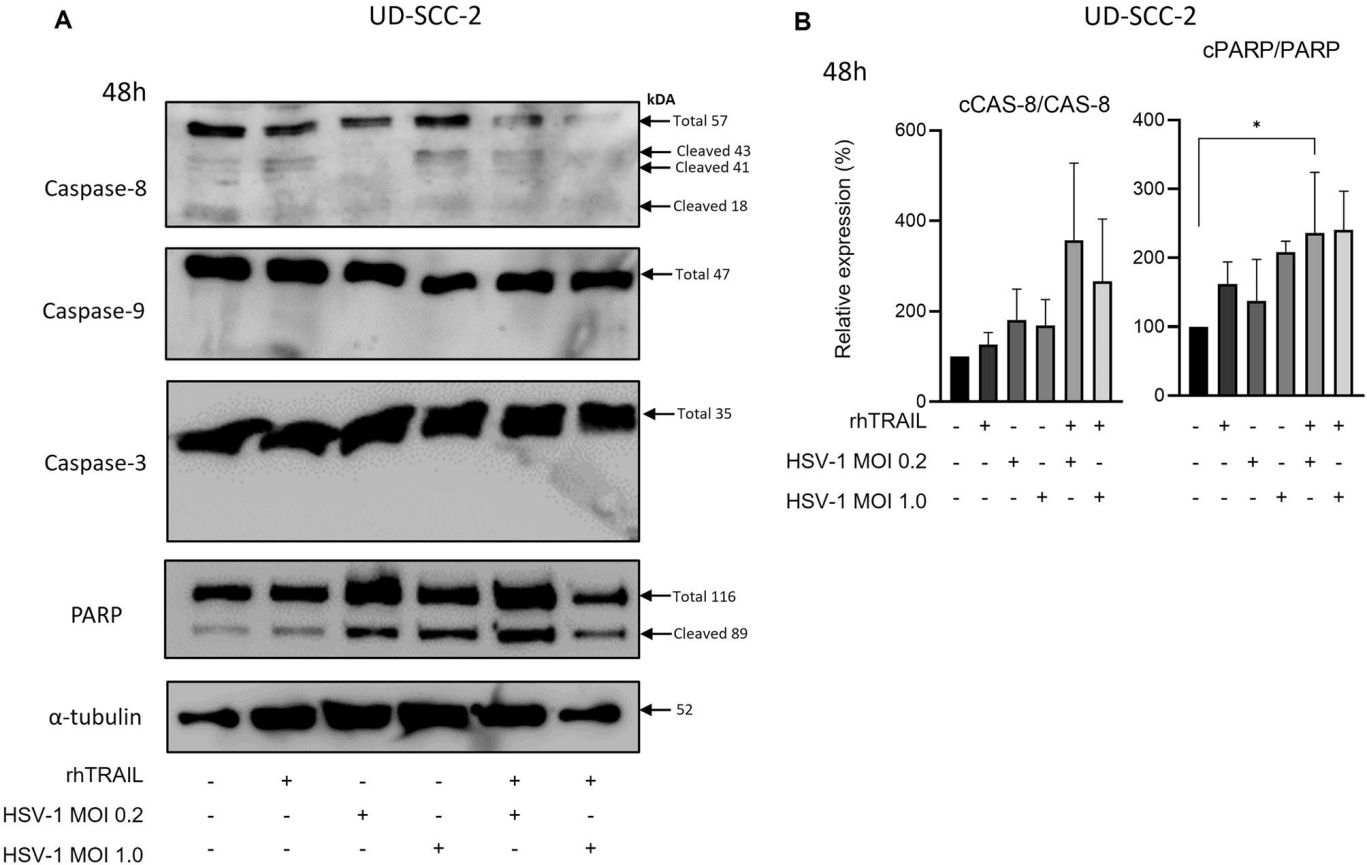
Expression analysis of apoptosis-associated proteins in the UD-SCC-2 cell line after 48 h of exposure with rhTRAIL ligand, WT HSV-1 and in combination. (**A**) and (**B**) detection and quantification of proteins after treatment. Results are expressed as mean percentage ± standard deviation of relative expression of cleaved protein relative to control (considering 100% expression) and normalized by the portion of total protein (non-cleaved). Data represent the average of two independent assays. The asterisks indicate statistical significance (*P < 0.05) in the comparison with the experimental groups by one-way ANOVA. Raw blots are presented in Supplementary Data 4.

After 48 h of treatment, we did not detect significant caspase-3, -8, or -9 cleavages in HCB289 or UD-SCC-2. However, we found PARP cleavages in both cell lines when rhTRAIL and HSV-1 were combined (Figs. 4A,B; 5A,B).

### rhTRAIL and HSV-1 induce apoptosis in HCB289 and UD-SCC-2 cell lines

**Figure 6:**
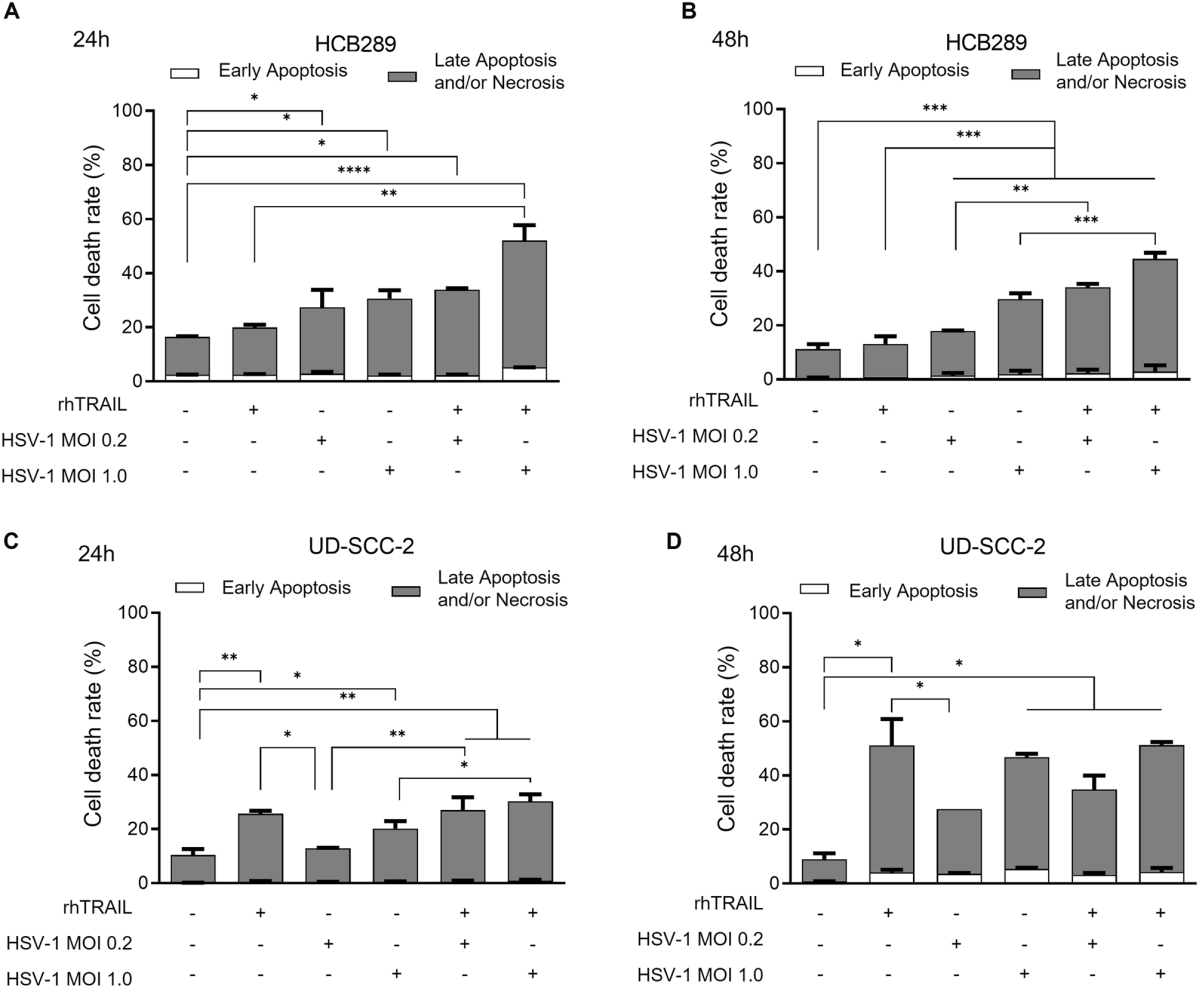
Flow cytometry apoptosis analysis after treatment with rhTRAIL ligand, WT HSV-1 and in combination. A and B) Analysis of HCB289 cell line after 24 and 48 h of treatment exposure. C and D) Analysis of UD-SCC-2 cell line after 24 and 48 h of treatment exposure. Results are expressed as mean percentage ± standard deviation of the cell death rate. Data represent the average of three independent assays. The asterisks indicate statistical significance (*P < 0.05; **P < 0.01; ***P < 0.001; ****P < 0.001) in the comparison with the experimental groups by one-way ANOVA.

Apoptosis flow cytometry analysis of rhTRAIL and HSV-1 treatments showed an increase in late apoptosis and necrosis in HCB289, 24 and 48 h post-treatment, mainly when combined with HSV-1 at MOI 1 of 52.12% and 44.62% respectively (Fig. 6A,B). Similarly, combined treatments in the UD-SCC-2 cell line showed an increase in late apoptosis and necrosis, but also the rhTRAIL monotherapy showed an induction of 25.7% and 51.15% for 24 and 48 h post-treatment, respectively (Fig. 6C,D).

We also compared the treatment conditions for each cell population (viable, necrotic, early apoptotic and late apoptotic) which demonstrated that the treatment was responsible for decreased viable cells detection as the cells entered the cell death process, mainly for rhTRAIL and HSV-1 presence for HCB289 (from 83.5%, negative control, to 47.4%, rhTRAIL + HSV-1 MOI 1.0 for 24 h posttreatment) and rhTRAIL alone (41.8% for 48 h) and in combination with the virus for UD-SCC-2 (42.3% at MOI 1.0 for 48 h posttreatment) (Supplementary Fig. S1).

The dot plots graphs generated by flow cytometer sample readings demonstrated a greater presence of double markers (annexin V-PE and 7AAD) by combining rhTRAIL and HSV-1 at MOI 1.0 for both HCB289 (50.9% for 24 h) and UD-SCC-2 (47.7% for 48 h) cell lines (Supplementary Figs. S2–S5).

### HSV-1 does not modulate the DR-5 receptor expression

**Figure 7:**
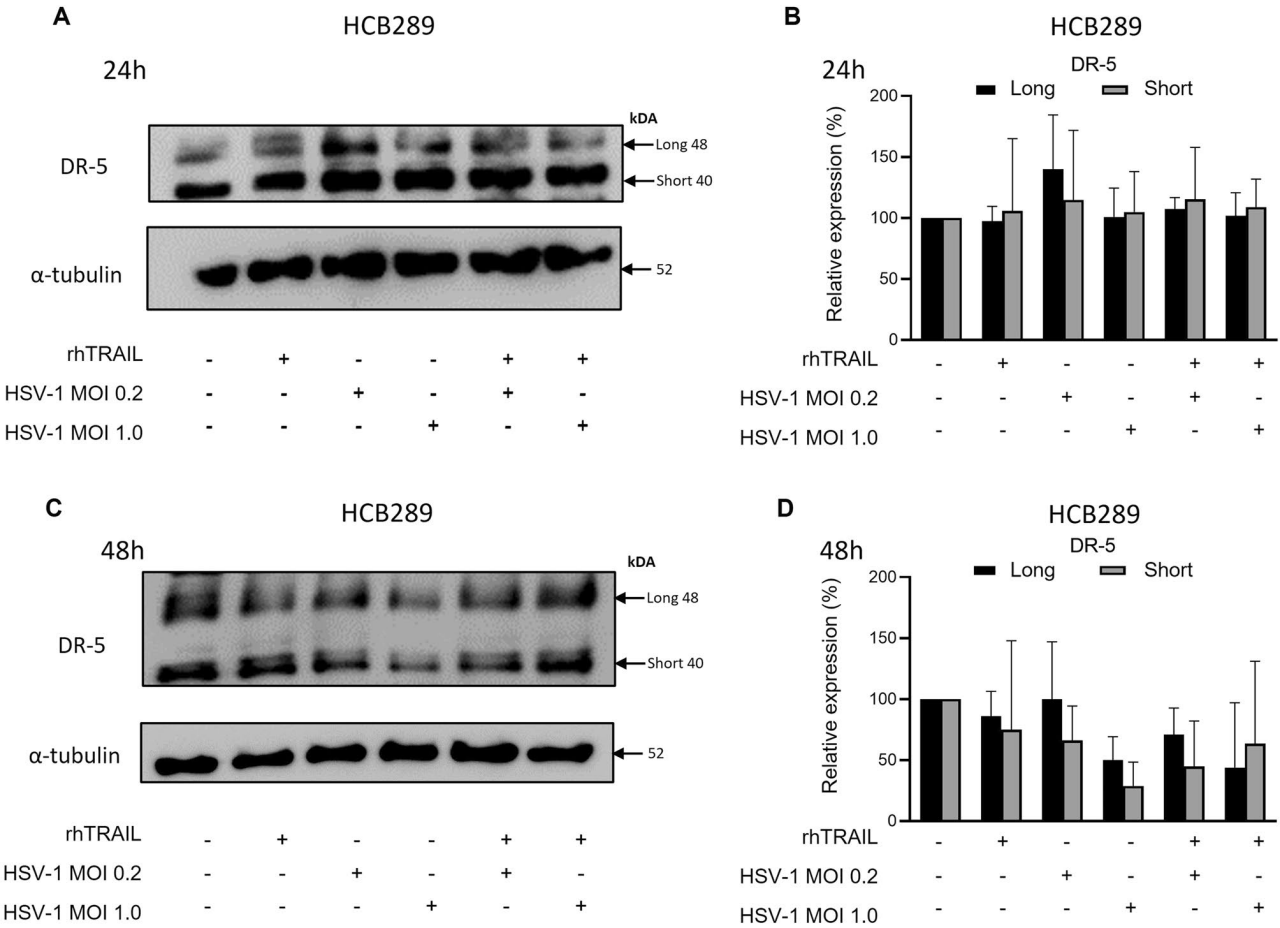
DR-5 expression analysis in the HCB289 cell line after treatment with rhTRAIL ligand, WT-HSV-1 and combination. (**A**) and (**B**) Detection and quantification of the receptor after 24 h of exposure treatment. (**C**) and (**D**) Detection and quantification of the receptor after 48 h of exposure treatment. Results are expressed as mean percentage ± standard deviation of relative expression of cleaved protein relative to control (considering 100% expression) and normalized by the protein α-tubulin. Data represent the average of two independent. Raw blots are presented in Supplementary Data 5.

**Figure 8:**
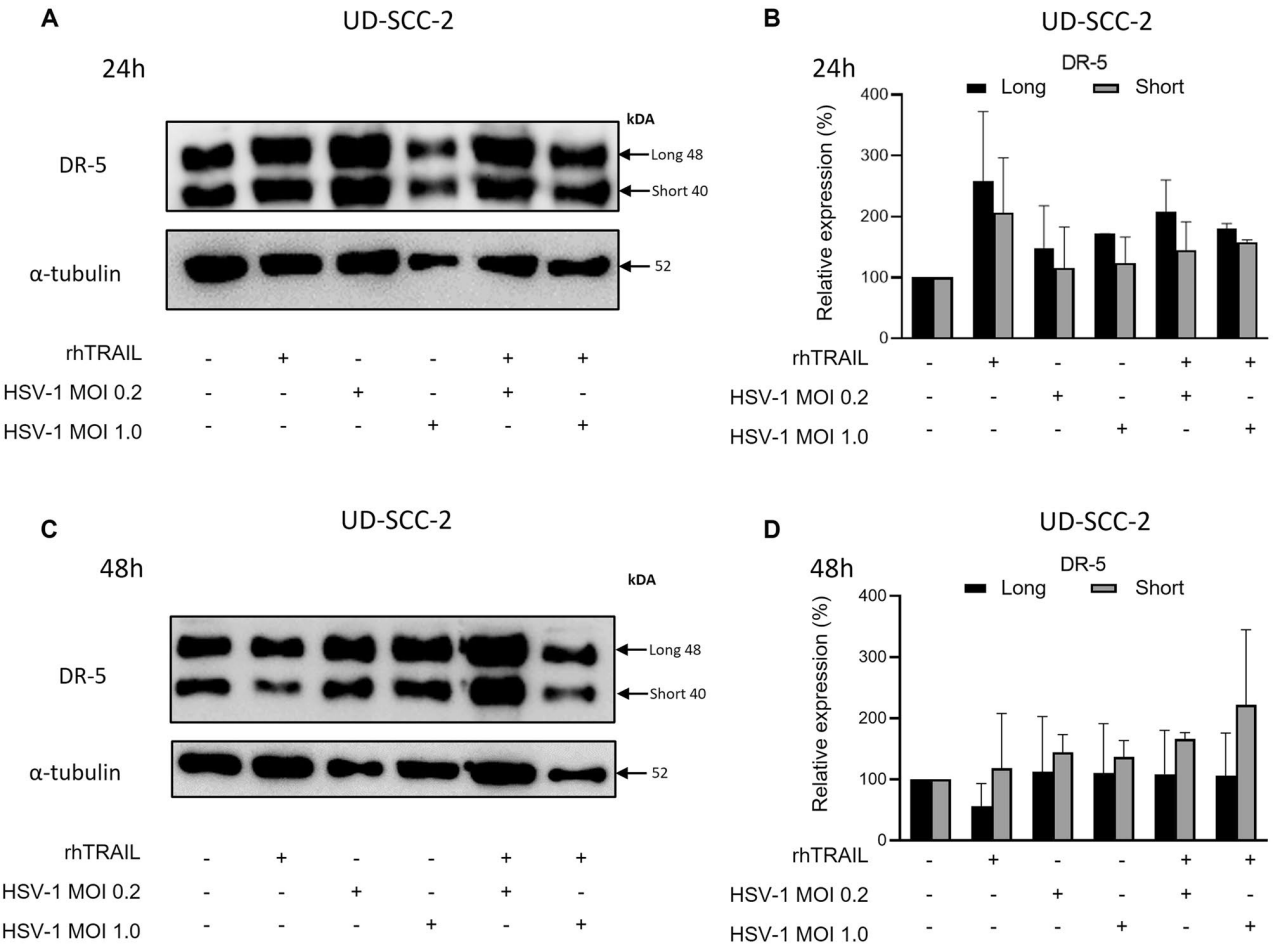
DR-5 expression analysis in the UD-SCC-2 cell line after treatment with rhTRAIL ligand, WT-HSV-1 and combination. (**A**) and (**B**) Detection and quantification of the receptor after 24 h of exposure treatment. (**C**) and (**D**) Detection and quantification of the receptor after 48 h of exposure treatment. Results are expressed as mean percentage ± standard deviation of relative expression of cleaved protein relative to control (considering 100% expression) and normalized by the protein α-tubulin. Data represent the average of two independent assays. Raw blots are presented in Supplementary Data 6.

Death receptor 5 (DR-5) expression was examined to investigate the possible mechanism responsible for modulating the apoptotic pathway triggered by the virus and the rhTRAIL ligand. After 24 and 48 h of the treatment with rhTRAIL, HSV-1 or both, no differences are observed in DR-5 receptor expression on HCB28 and UD-SCC-2 cell lines (Figs. 7, 8). These results suggest that other cellular mechanisms may be responsible for the biological effects reported in the present study.

## Discussion

This study explores an advanced head and neck cancer treatment using wild-type HSV-1 infection in combination with the rhTRAIL protein. TRAIL ligand can be naturally released as a soluble form by proteolysis, mostly promoting apoptosis in tumor cells^25, 26^. The soluble form of TRAIL is well tolerated by non-cancer cells; and traces can be found in the healthy adult plasma (~ 100 pg/mL)^27, 28^. Moreover, its cognate functional cell death receptors, DR4 and DR5, are ubiquitous expressed in several tumor cell types^17^. Several clinical studies are based on recombinant human TRAIL protein (rhTRAIL) and agonist antibodies to TRAIL receptors. However, none of these molecules alone or combined with other therapeutic agents have acquired the efficacy expected in clinical trials^20, 25^.

Currently, many therapies use gene therapy to target the TRAIL gene and combinatorial use of this ligand with various other molecules, such as chemotherapies^24, 29–32^. Griffith and colleagues showed, using a TRAIL-expressing adenovirus, that the rapid expression of TRAIL protein in tumor cells triggered apoptosis via caspase-8 activation^29^. A similar response was observed in our study, where rhTRAIL in monotherapy or combination with HSV-1 virus induced apoptosis by activating caspase-8 cleavage in the UD-SCC-2 cell line.

Jeong and Yoo treated 14 tumor cell lines and a colorectal cancer murine model with a TRAIL/ANGPT1-co-expressing oncolytic vaccinia virus, showing that the dual expression of these molecules was effective in inducing apoptosis and inhibiting tumor growth in the in vivo assays, followed by caspase 3 and CD8 expression in tumors^30^. Likewise, we showed by flow cytometry analysis that the combination of HSV-1 virus and rhTRAIL treatments induced apoptosis in the HCB289 and UD-SSC-2 cell lines . Besides, TRAIL-treated UD-SCC-2 cell line also showed induction of late apoptosis/necrosis as monotherapy. These results demonstrate the potential of combining therapeutic approaches to attack tumor cells.

In the same way, Gao and colleagues demonstrated a synergistic effect when treating colorectal cancer cell lines with a TRAIL-expressing oncolytic adenovirus in combination with radiotherapy^31^. Authors showed that the armed oncolytic virus triggered apoptosis through caspase-3 and caspase-8 cleavage in the SW480 cell line, mainly when combined with radiotherapy^31^. In our work, we showed apoptosis involvement by PARP cleavage when HCB289 and UD-SCC-2 cell lines were treated with the combination of rhTRAIL and HSV-1 virus.

Moreover, as shown by Hu and co-workers, when treating lung tumor cell lines with a poxvirus-derived oncolytic virus expressing TRAIL, cells undergone apoptosis, while the parental poxvirus non-expressing TRAIL triggered RIP3-dependent necrosis^32^. Although in our study, we did not evaluate the expression of RIP1/RIP3, we observed cell- and treatment-specific cell death response profiles. Thus, HSV-1 virus induced caspase-9 cleavage in the HCB289 cell line, while caspase-8 cleavage was observed when rhTRAIL was inoculated, either alone or in combination with the virus.

In another study, Kaoru and colleagues tested in glioblastoma (GBM) and stem cell-derived (GSC) cell lines the biological effect of an HSV-1-based oncolytic virus (oHSV), TRAIL, and a recombinant virus with TRAIL expression (oHSV-TRAIL)^24^. They observed by western blotting that cleavage of PARP was not detected by either TRAIL or oHSV as monotherapy in glioblastoma cells, indicating that oHSV-mediated cell death is primarily not dependent on caspase/PARP activation^24^. In contrast, oHSV-TRAIL showed cleavage of caspase-8, -9, and PARP and induction of apoptosis in the cell lines resistant to TRAIL or oHSV alone^24^. The authors inferred that oncolytic virus-based gene therapies might be feasible for different tumor types with different molecular profiles^24^. Our study also did not detect PARP cleavage by TRAIL monotherapy, but it was possible to detect PARP cleavage for the HCB289 cell line in the presence of HSV-1 virus at both concentrations (MOI 0.2 and MOI 1.0). This highlights that the HCB289 cell line resistant to TRAIL monotherapy could be induced apoptosis by the presence of the virus.

Although rhTRAIL was described as able to induce apoptosis mainly on tumor cells^15^, we showed that the HaCaT cell line also showed a reduction in cell viability. Although often used as a normal control, it is worth mentioning that HaCaT is an aneuploid immortalized keratinocyte cell line, thus a limitation of our work. Regardless of this fact, the main objective of this proof-of-concept work was to evaluate the combined effect of rhTRAIL and HSV-1 oncolysis against HNSCC. In this regard, authors understand that the use of a wild-type HSV-1 virus could never be used in a clinical trial setup, and we point this out as a limitation of the study. To this end, additional experiments must be conducted to further establish proper attenuation of the therapeutic approach.

To our knowledge, this is the first study combining HSV-1 virus and TRAIL as therapy for HNSCCs, showing that the combination potentiates the viability reduction through cell death induction. Our results open new therapeutic approaches for HNSCC patients, which should be explored in further studies.

## Material and methods

### Cell lines

Vero cells (ATCC #CCL-81, Manassas, VA, USA) were used to produce the wild-type HSV-1 virus. Four HNSCC-derived cell lines (UD-SCC-2, gently donated from Henning Bier; UM-SCC-47, Merck Millipore, Burlington, MA, USA; UM-SCC-104, Merck Millipore, Burlington, MA, USA; FaDu, ATCC #HTB-43, Manassas, VA, USA) were used in the experiments. The immortalized human keratinocytes cell line, HaCat (gently donated from Rui M. Reis) was used as a non-tumoral control. All cell lines were maintained in DMEM medium supplemented with 10% fetal bovine serum (FBS, Thermo Fisher Scientific, Waltham, MA, USA) and 1% penicillin/streptomycin (Sigma-Aldrich, San Luis, MO, USA), in a humidified incubator at 37 °C with 5% CO_2_. Cell lines authentication was performed by the Department of Molecular Diagnostics, Barretos Cancer Hospital. Genotyping confirmed the identity of all cell lines, as previously described by Oliveira et al^33^. Moreover, all cell lines were tested for mycoplasma through the MycoAlert™ Mycoplasma Detection Kit (Lonza Bioscience, catalog no. LT07-318, Morrisville, NC, USA), following the manufacturer’s instructions.

### HSV-1 viral production

The virus used in this work was an HSV-1 virus (strain 17), cloned as a bacterial artificial chromosome^34^ (gently donated from David A. Leib, Geisel School of Medicine at Dartmouth, Department of Microbiology and Immunology). Viral amplification was performed at MOI 0.01 in Vero cells in M199 medium supplemented with 1% FBS (Thermo Fisher Scientific, Waltham, MA, USA) and 1% penicillin/streptomycin (Sigma-Aldrich, San Luis, MO, USA), in a humidified incubator at 37 °C with 5% CO_2_. Viral particles were collected 72 h post-infection. Intracellular viral particles were extracted by three cycles of freeze and thaw, followed by three cycles of sonication. Cellular debris were eliminated by centrifugation at 600 × *g* for 20 min (4 °C), and supernatants were collected and stored at –80 °C until required.

### Virus titration and MOI calculation

Viral titration were performed by infinite dilution in Vero cells^35^. A serial dilution was perfomed to determinate the number of plaque-forming unit per mL (pfu / mL) by duplicate into 12-well. We maintained the plate under shaker (ProBlot™ 25 Economy Rocker, Labnet, Edison, NJ, USA) for one hour and thirty minutes with 400 uL volume of infection per well and after the infection time, the volume per well was completed to 800 µL, with 200 µL of M199 medium and 200 µL of CMC 1% (carboxymethylcellulose, diluted in PBS 1x). The plate was in a humidified incubator at 37 °C with 5% CO_2_. After 72 h, the wells were stained with 1 mL crystal violet (0.5%) for 5 min and the lysis plates counted. The virus titer was determined by concentration in pfu/mL of our HSV-1 virus stock was calculated by considering the mean of the duplicate for the number of plaques counted from the given dilution with the following Eq. (1):

1 $${\text{pfu}}/{\text{mL }} = \frac{{{\text{Average }}\;{\text{of }}\;{\text{plaques}}}}{{{\text{Dilutation}}\;{\text{factor}}\; \times \;{\text{Volume}}\; {\text{of}}\; {\text{diluted}}\;{\text{ virus}}\; {\text{added}}\; {\text{to}}\; {\text{the}}\; {\text{plate}}}}$$

To determinate the volume of virus to infect our cells according to the MOI value, we used the following Eq. (2):

2 $${\text{MOI}} = \frac{{{\text{Virus}}\; {\text{Titer}}\; \times \;{\text{Virus}}\;{\text{Volume}}}}{{{\text{Total}}\;{\text{Cell}}\;{\text{Number}}}}$$

### Viability cellular assays

Cell viability assay was performed using the Cell Titer 96 Aqueous One Solution Cell Proliferation Assay (MTS assay- Promega Corporation, catalog no. G3581, Madison, WI, USA), following the manufacturer´s recommendations. Briefly, HNSCC cells were seeded into 96-well plates at 5 × 10^3^ cells per well in triplicate. Cells were treated with rhTRAIL (#375-TL, R&D Systems, Minneapolis, MN, EUA) at 100 ng/mL, HSV-1 MOI 0.2 and/or HSV-1 MOI 1. PBS was used as negative control. MTS assay was performed 48 and 72 h post-treatments. Absorbance was measured in a microplate reader (Varioskan Flash, Thermo Fisher Scientific, Waltham, MA, USA) at a wavelength of 490 nm.

### Western blot

Cells were scrapped and resuspended in lysis buffer (50 mM of Tris (pH 7.6–8. 0), 150 mM NaCl, 5 mM of EDTA, 1 mM of Na_3_VO_4_, 10 mM of NaF, 10 mM de sodium pyrophosphate, 1% NP-M de EDTA e 1 mM de PMSF). Cell debris were eliminated by centrifugation at 14,000 × *g* for 15 min (4 °C) and proteins assayed by SDS-PAGE (acrylamide/bis-acrylamide) electrophoresis. Nitrocellulose membrane – transferred proteins (Amersham ProtaN supported 0.45 µm NC, GE Healthcare, Chicago, IL, EUA) were blocked for 1 h in 5% low-fat milk + TBS-T (10 mM tris–HCl pH 7.4 + 0.9% NaCl, 0.2% Tween)] and incubated with the primary antibodies anti-PARP (1:1000; Cell Signaling Technology; #9542, Danvers, MA, USA), anti-DR-5 (1:1000; Cell Signaling Technology; #69,400, Danvers, MA, USA), anti-caspase-9 (1:1000; Cell Signaling Technology; #9502, Danvers, MA, USA), anti-caspase-8 (1:1000; Cell Signaling Technology; #9746S, Danvers, MA, USA), anti-caspase-3 (1:1000; Cell Signaling Technology; #9662, Danvers, MA, USA), anti-α-tubulin (1:1000; Cell Signaling Technology; #3873, Danvers, MA, USA) were incubated overnight at 4 °C. After incubation, membranes were incubated with the secondary antibodies anti-rabbit IgG HPR (1:5000; Cell Signaling Technology; #7076, Danvers, MA, USA) or anti-mouse IgG HPR (1:5000); Cell Signaling Technology; #7074, Danvers, MA, USA) as required. Detection was performed using Amersham ECL Prime Western Blotting Detection Reagent (GE Healthcare, Chicago, IL, EUA) or SuperSignal West Femto (Thermo Fisher Scientific, catalog no. 34096, Waltham, MA, USA) on the ImageQuant LAS 4000 mini (GE Healthcare, Chicago, IL, USA).

### Apoptosis assay by flow cytometry

Flow cytometry analysis was done using PE Annexin V apoptosis detection kit l (BD Biosciences, Haryana, HR, USA), following manufacturer’s instructions. Briefly, cells were seeded into 12-well plates at 2.5 × 10^5^ per well. After treatment, trypsinized cells were resuspended in 500 µL of Binding Buffer 1X and subsequently stained with annexin V-PE and 7AAD. Stained cells were evaluated with the BD Accuri C6 flow cytometer (BD Biosciences, Haryana, HR, USA) and analyzed using the FlowJo software (BD Biosciences, Haryana, HR, USA; version 10.8.1 for Windows).

### Statistical analysis

Statistical analysis was done using GraphPad Prism software (version 8.4.3 for Windows). Cell viability was analyzed by non-normal distribution Kruskal–Wallis test. Western blotting and apoptosis assay were analyzed by one-way ANOVA. All data are shown as the mean ± standard deviation (SD), in duplicate and triplicate assays. Statistical significance for *p* value was set at < 0.05 (*p < 0.05, ** *p* < 0.01, ***p < 0.001, ****p < 0.0001).

### Supplementary Information

Supplementary Information 1.

Supplementary Information 2.

## Data Availability

All data needed to evaluate the conclusions in the paper are present in the paper and/or the Supplementary Materials. Any additional information are available from the corresponding author.
