# Supplementary material for: Combined effect of the pro-apoptotic rhTRAIL protein and HSV-1 virus in head and neck cancer cell lines
Source: Sci Rep. 2023 Oct 21;13:18023. doi: 10.1038/s41598-023-44888-9 (PMC10590400; doi:10.1038/s41598-023-44888-9)
Supplement: Supplementary file 1 — Supplementary Information 1. [file 41598_2023_44888_MOESM1_ESM.pdf]

Supplementary figures

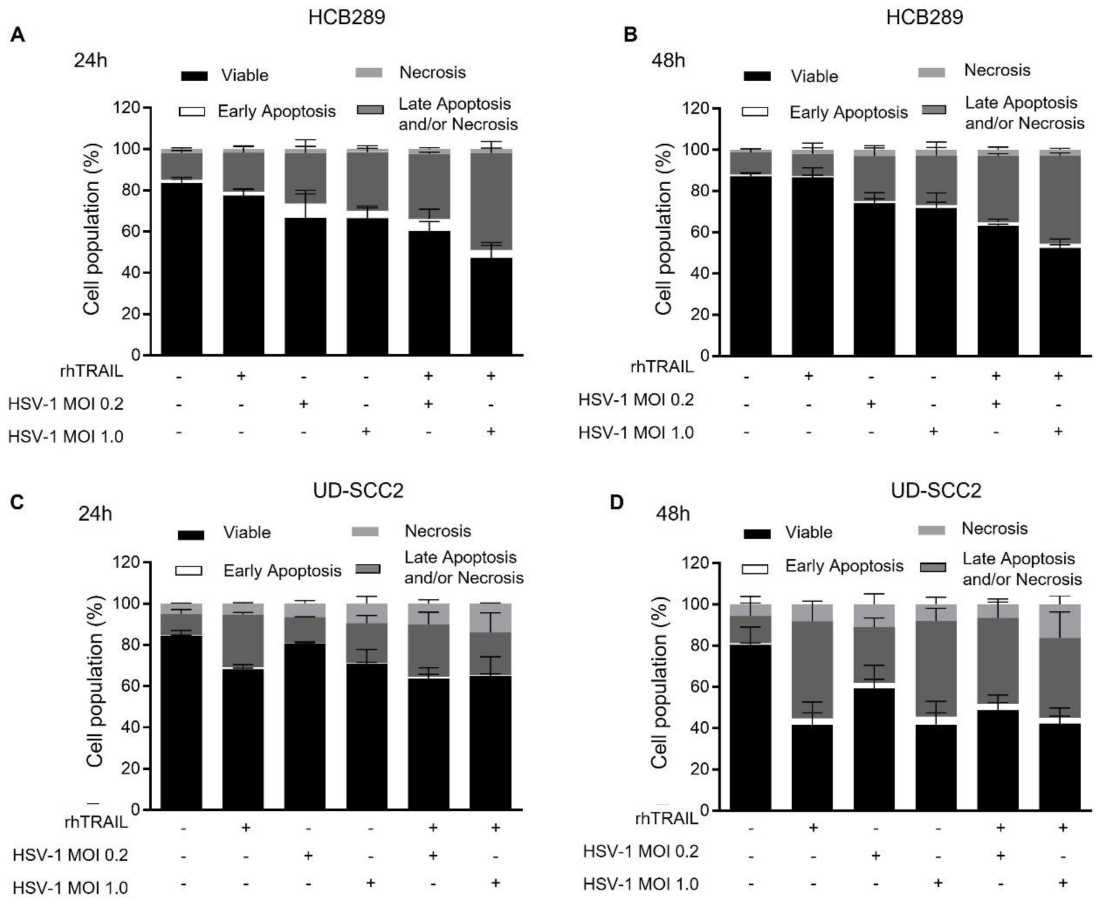

Figure S1. Comparison of viable cells, in necrosis, in early apoptosis and in late apoptosis (and/or necrosis) after treatment with rhTRAIL ligand, HSV-1 WT and in combination by flow cytometry. **A** and **B)** Analysis of HCB289 cell line after 24 and 48 hours of treatment exposure. **C** and **D)** Analysis of UD-SCC-2 cell line after 24 and 48 hours of treatment exposure. Results are expressed as mean percentage ± standard deviation of percent total cell population. Data represent the average of three independent assays performed in triplicate.

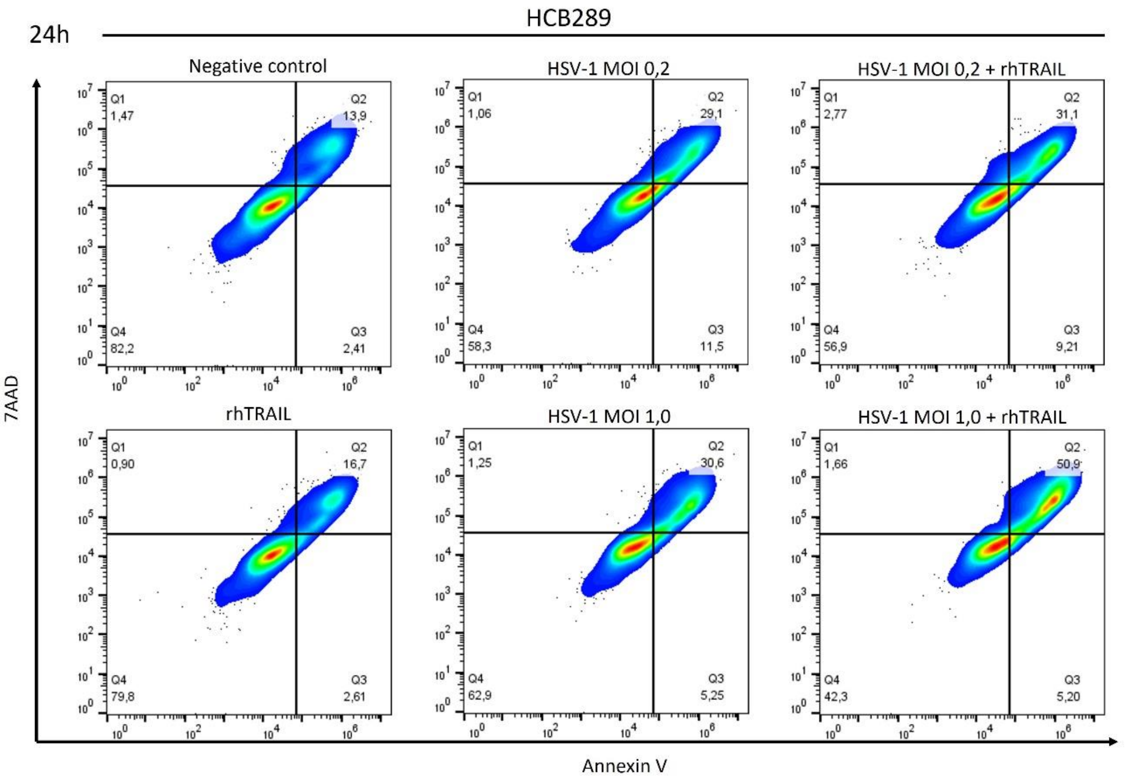

Figure S2. Dot plot graphs of flow cytometry analysis of apoptosis assay in the HCB289 cell line after 24 hours of exposure with rhTRAIL ligand, WT HSV-1 and in combination. Q1: Necrosis; Q2: Late apoptosis (and/or necrosis); Q3: Early apoptosis; Q4: Viable. Data represent the most representative assay of a biological triplicate

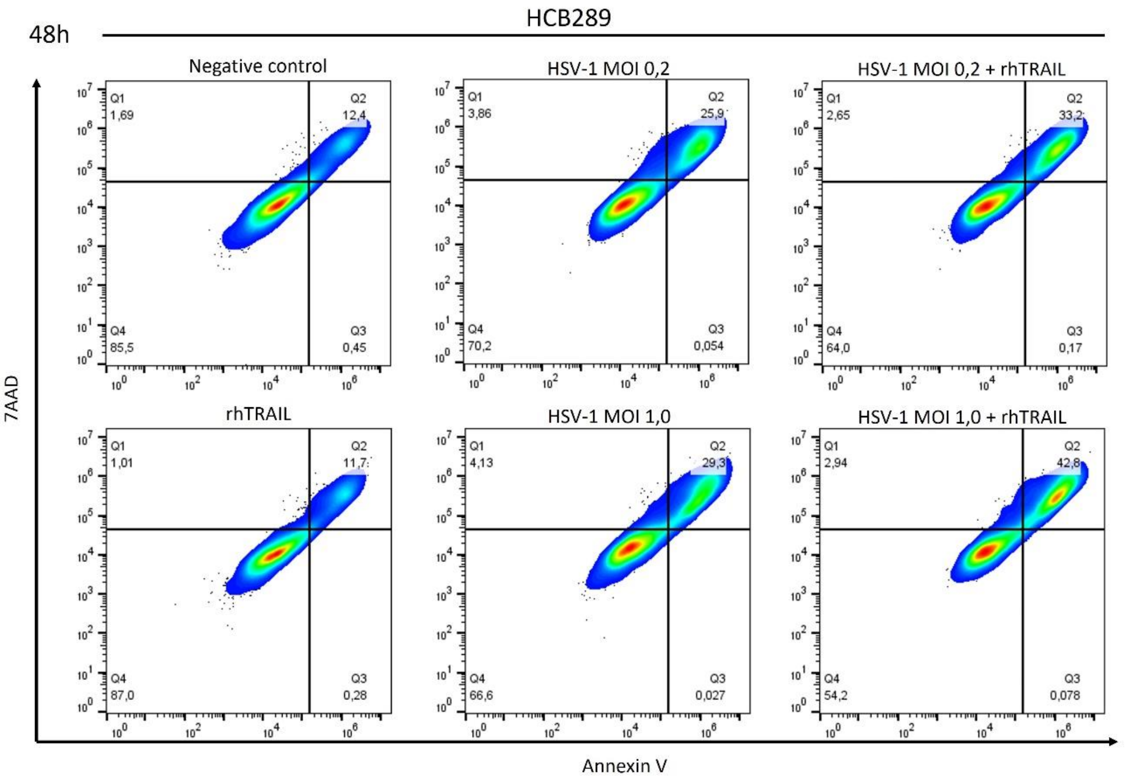

Figure S3. Dot plot graphs of flow cytometry analysis of apoptosis assay in the HCB289 cell line after 48 hours of exposure with rhTRAIL ligand, WT HSV-1 and in combination. Q1: Necrosis; Q2: Late apoptosis (and/or necrosis); Q3: Early apoptosis; Q4: Viable. Data represent the most representative assay of a biological triplicate

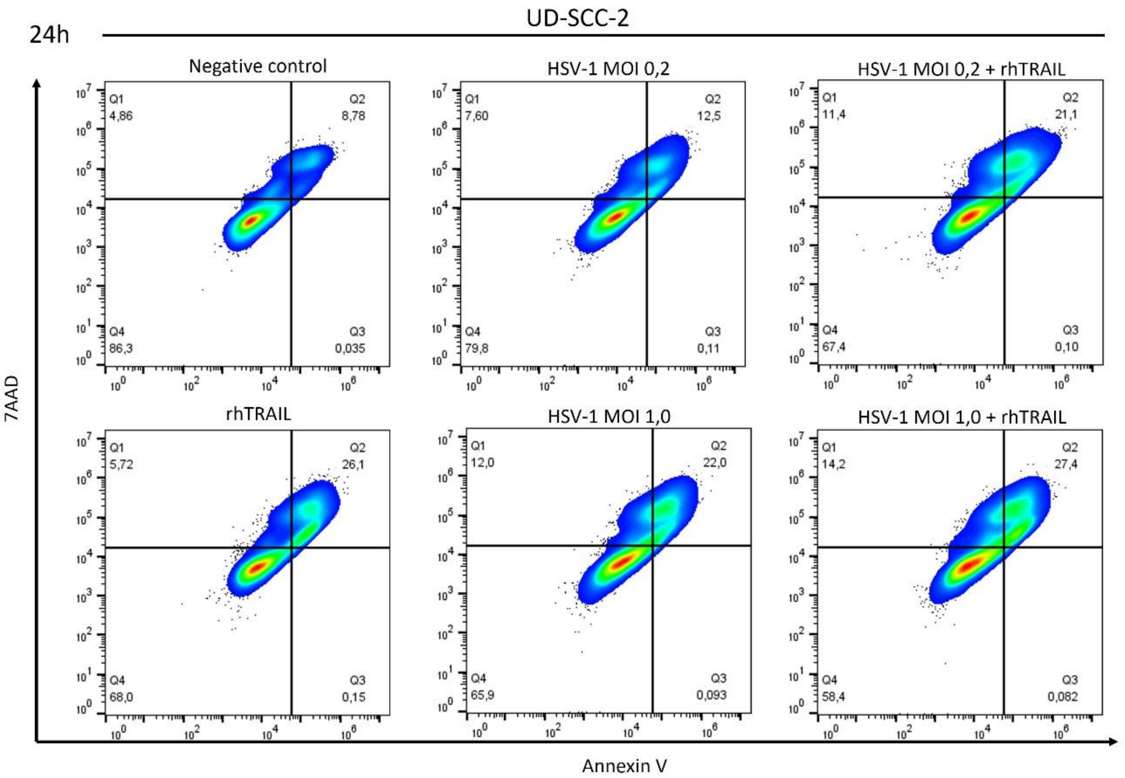

Figure S4. Dot plot graphs of flow cytometry analysis of apoptosis assay in the UD-SCC-2 cell line after 24 hours of exposure with rhTRAIL ligand, WT HSV-1 and in combination. Q1: Necrosis; Q2: Late apoptosis (and/or necrosis); Q3: Early apoptosis; Q4: Viable. Data represent the most representative assay of a biological triplicate

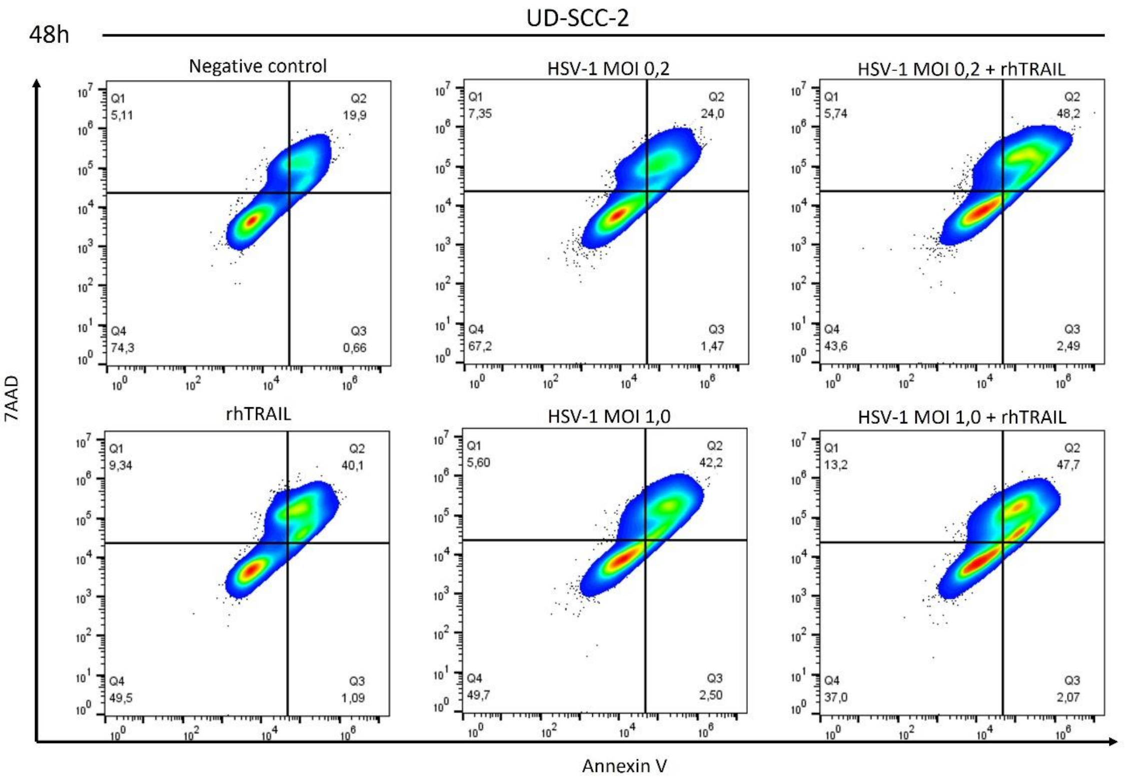

Figure S5. Dot plot graphs of flow cytometry analysis of apoptosis assay in the UD-SCC-2 cell line after 48 hours of exposure with rhTRAIL ligand, WT HSV-1 and in combination. Q1: Necrosis; Q2: Late apoptosis (and/or necrosis); Q3: Early apoptosis; Q4: Viable. Data represent the most representative assay of a biological triplicate
