# Supplementary material for: Combined effect of the pro-apoptotic rhTRAIL protein and HSV-1 virus in head and neck cancer cell lines
Source: Sci Rep. 2023 Oct 21;13:18023. doi: 10.1038/s41598-023-44888-9 (PMC10590400; doi:10.1038/s41598-023-44888-9)
Supplement: Supplementary file 2 — Supplementary Information 2. [file 41598_2023_44888_MOESM2_ESM.pdf]

Supplementary data

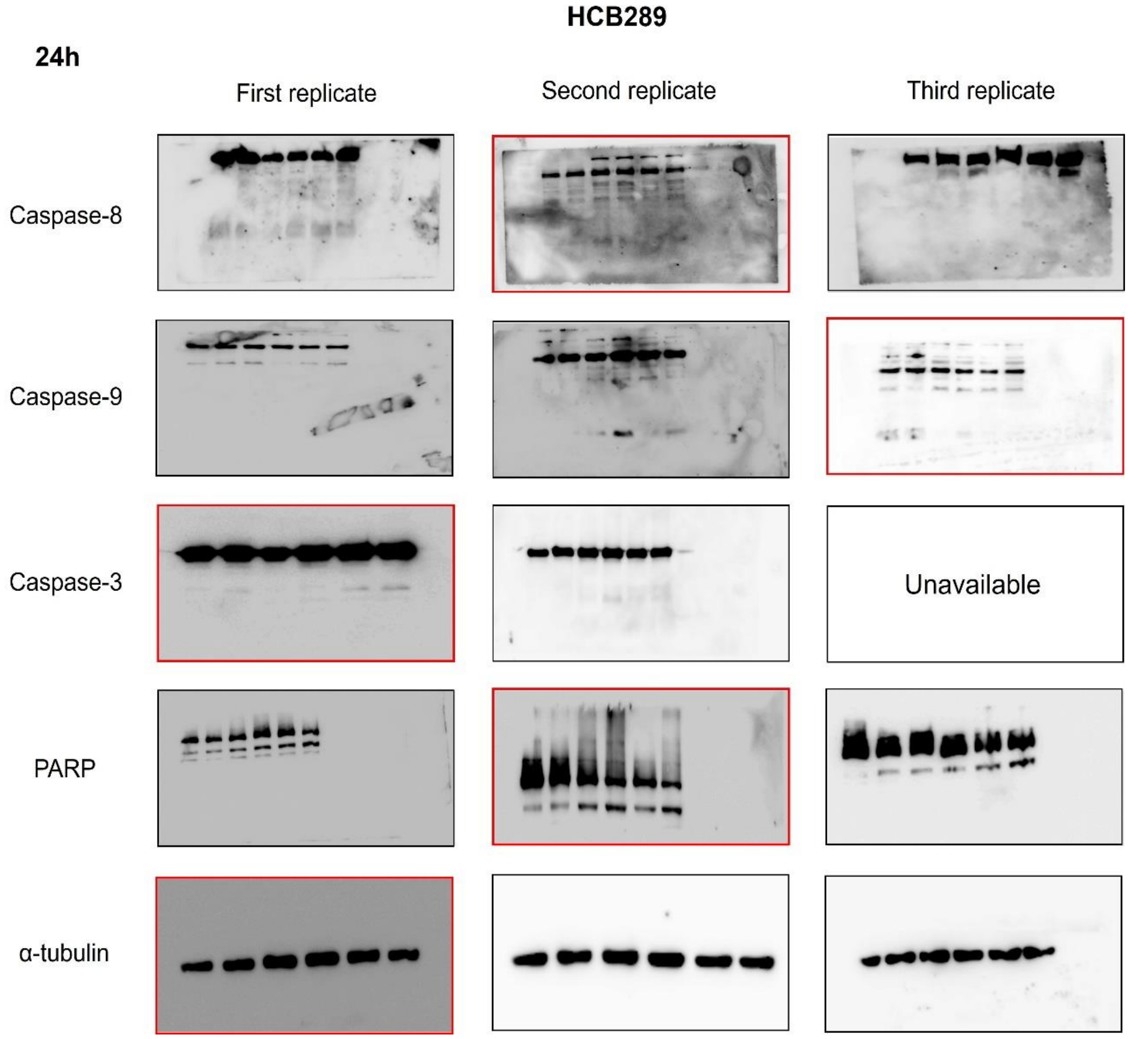

Supplementary Data 1. Raw western blot images of Figure 2A. Images used in the manuscript are marked with a red outline.

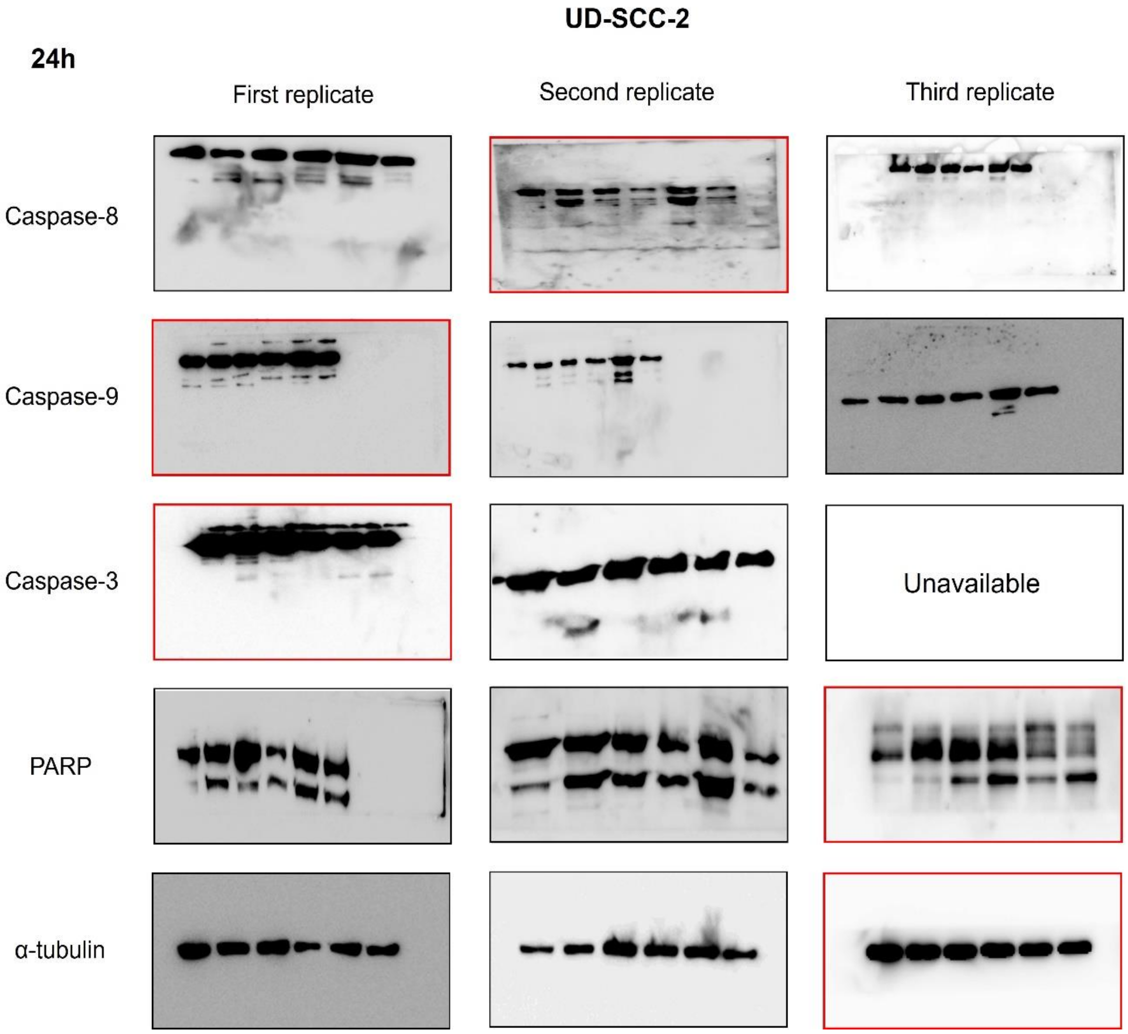

Supplementary Data 2. Raw western blot images of Figure 3A. Images used in the manuscript are marked with a red outline.

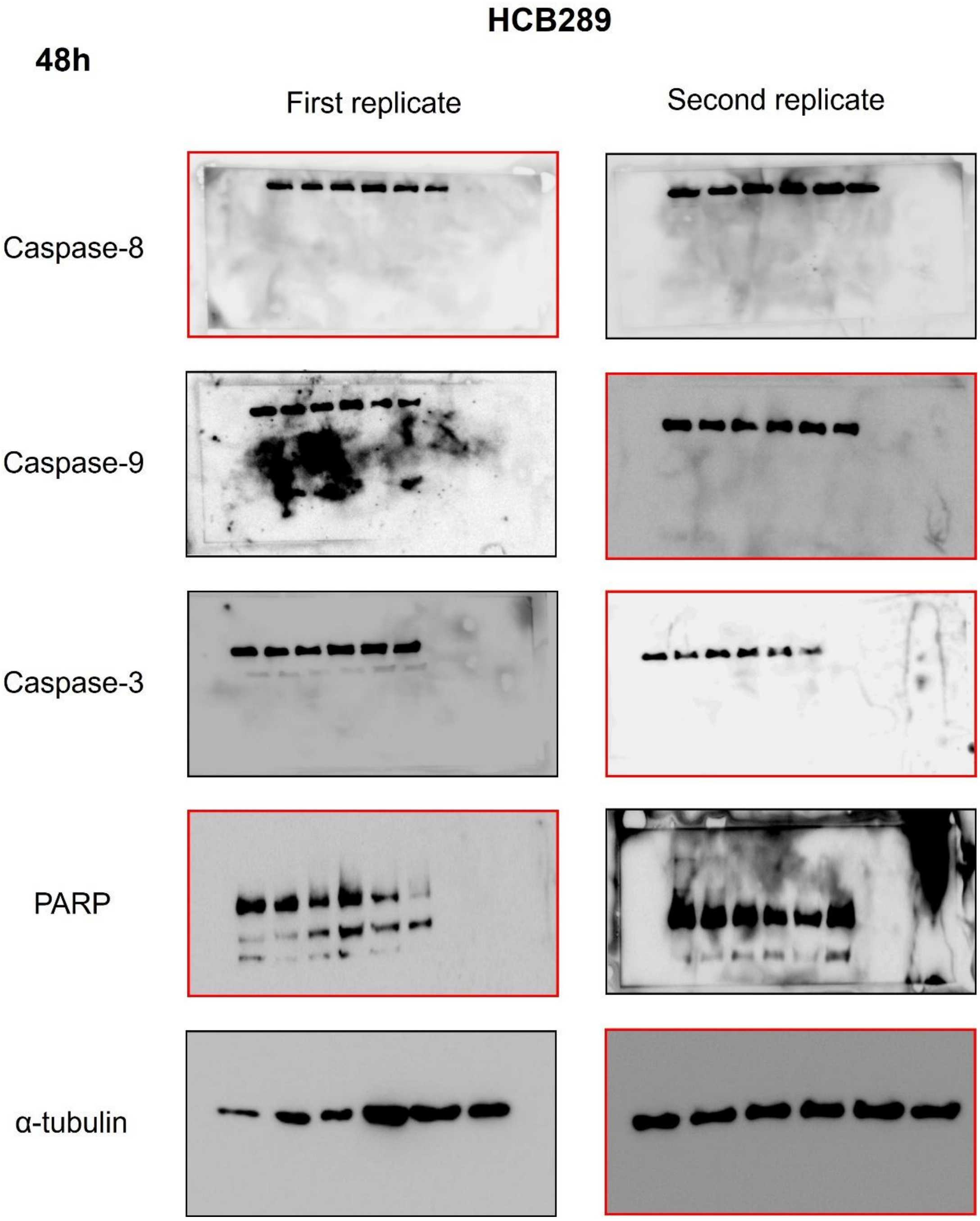

Supplementary Data 3. Raw western blot images of Figure 4A. Images used in the manuscript are marked with a red outline.

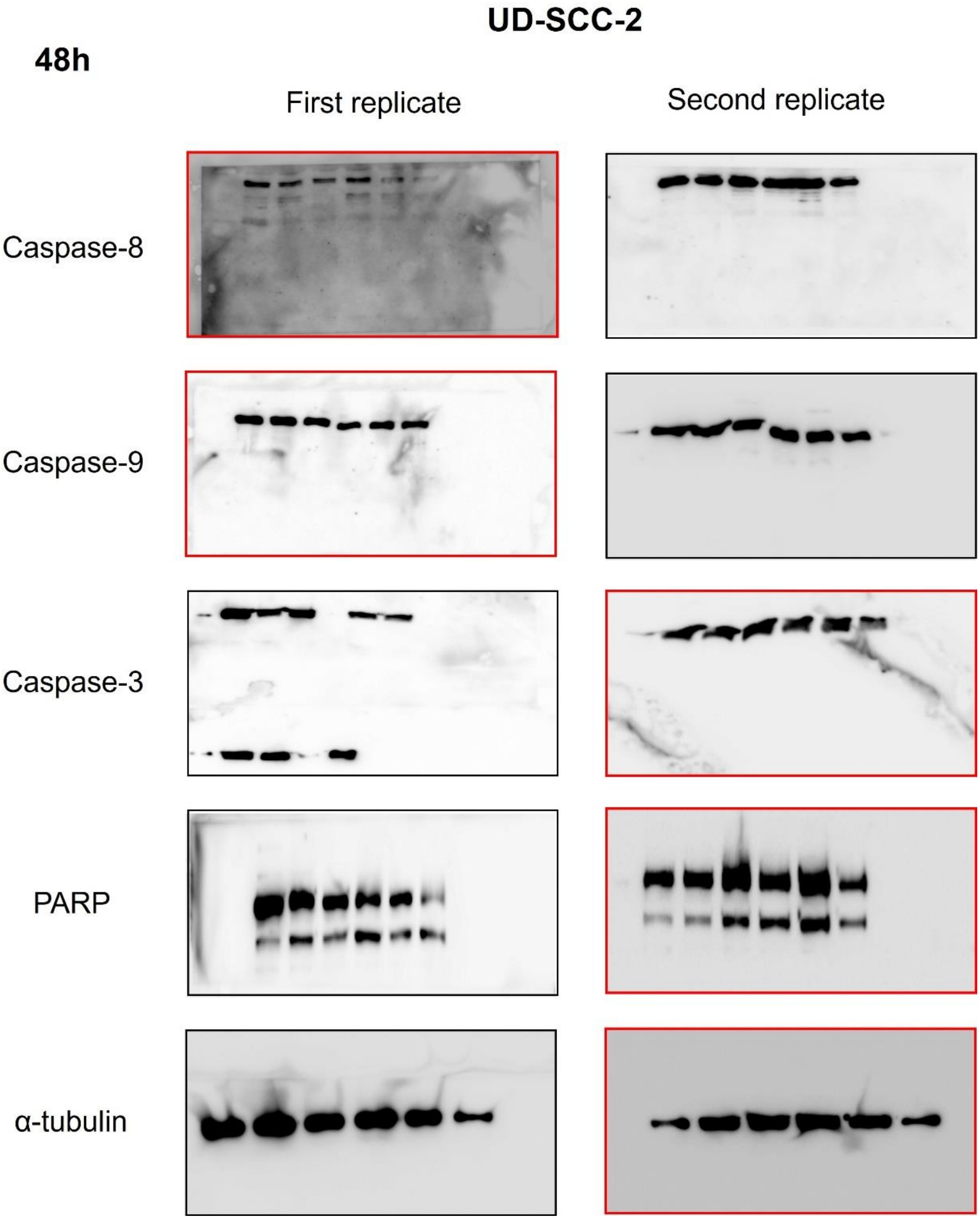

Supplementary Data 4. Raw western blot images of Figure 5A. Images used in the manuscript are marked with a red outline.

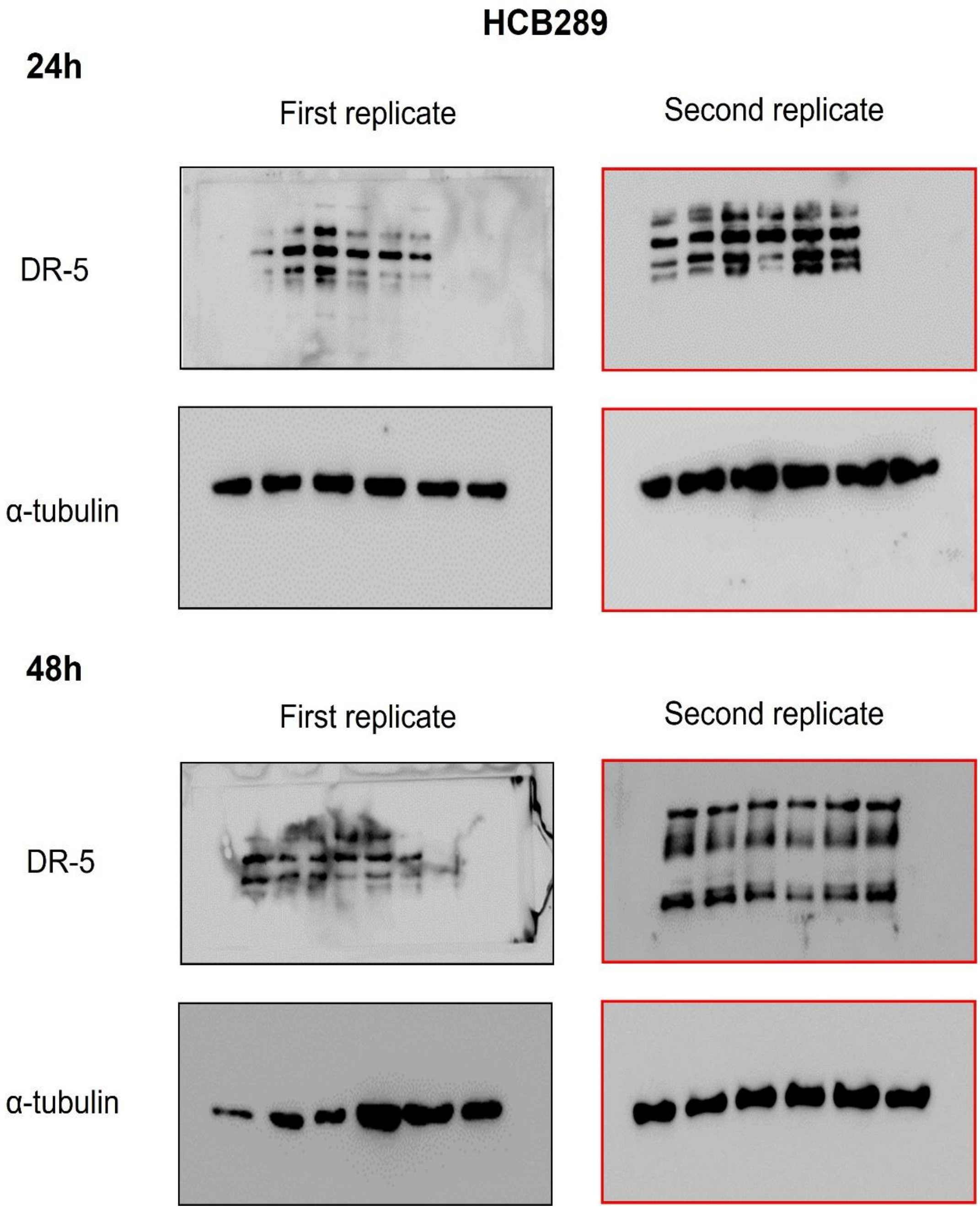

Supplementary Data 5. Raw western blot images of Figure 7A and 7C. Images used in the manuscript are marked with a red outline.

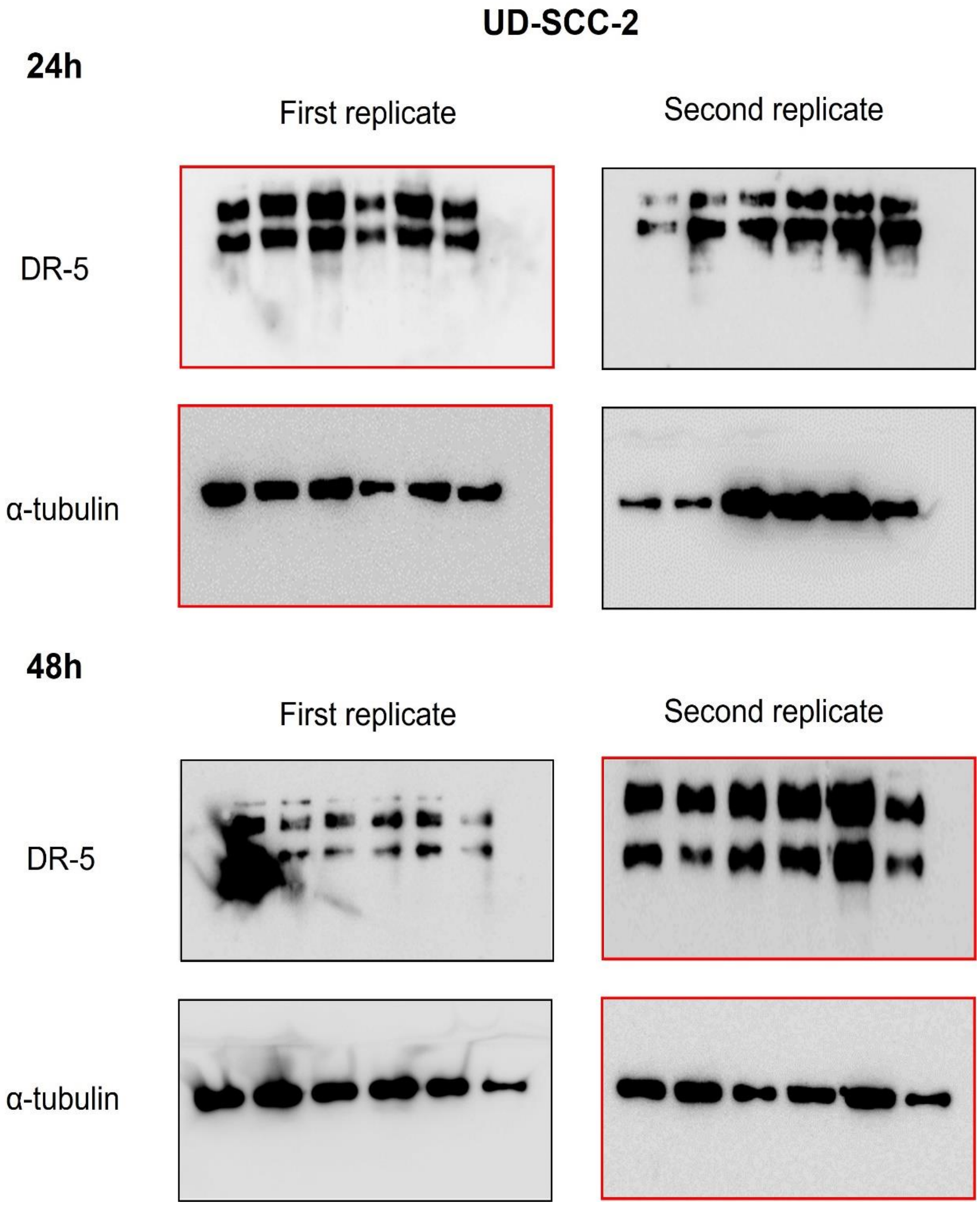

Supplementary Data 6. Raw western blot images of Figure 8A and 8C. Images used in the manuscript are marked with a red outline.
